# Effects of saddle tilt and stirrup length on the kinetics of horseback riders

**DOI:** 10.7717/peerj.14438

**Published:** 2022-12-05

**Authors:** Marc Elmeua González, Nejc Šarabon

**Affiliations:** 1 Faculty of Health Sciences, University of Primorska, Izola, Slovenia; 2 University of Primorska, Faculty of Health Sciences, Izola, Slovenia; 3 Laboratory for Motor Control and Motor Behaviour, S2P, Science to Practice, ltd., Ljubljana, Slovenia

**Keywords:** Equitation sciences, Horse riding biomechanics, Saddle, Stirrups, Reins

## Abstract

**Background:**

How the modification of saddle fitting parameters in horse riding affects rider’s kinetics is very uncertain. The aim of this study is to describe how manipulating the two main adjustments that an end-user is likely to perform (saddle tilt and stirrup length) affects the biomechanics of a horse rider on a living horse.

**Methods:**

Eleven showjumpers volunteered to take part in this study. Each participant performed a 120-strides standardization trial at trot and canter, with 0° saddle tilt and stirrup length that would position the rider’s knee at 90°. Following the standardization trial, four interventions were performed, which consisted of 60 strides with 60 mm shorter stirrups, 60 mm longer stirrups, 4° forward tilted saddle and 4° backward tilted saddle. Stirrup and rein tension forces were measured with tension loadcells. A symmetry index was calculated. Acceleration was measured with inertial measuring units at the helmet and back of the rider and shock attenuation was calculated.

**Results:**

Shortening the stirrups and adjusting saddle tilt significantly enhanced shock attenuation at canter and increased force on the stirrups at trot and canter (*p* < 0.05). Lowering the stirrups reduced rein tension forces (*p* = 0.01). At trot, adjusting saddle tilt and stirrup length enhanced symmetry index on the bit (*p* < 0.05). These results allowed for general guidelines to be proposed, although individualization became an evident part of any saddle setup design due to a high inter-subject variability.

## Introduction

Lower back pain is the most common chronic injury experienced by horse riders (Kraft et al., 2009; Feucht & Patel, 2010; Greve & Dyson, 2013; Lewis & Kennerley, 2017; Cejudo et al., 2020), and it has been linked to poor postural control and alignment at the pelvic level (Nevison & Timmis, 2013; Hobbs et al., 2014). Despite this, adjusting saddles to fit riders has not been a priority in the scientific literature of show jumping equestrianism. Instead, authors have focused on how manipulating saddles to fit their horses can influence the animal’s performance and top line health (Kotschwar, Baltacis & Peham, 2010; Greve & Dyson, 2013; Dyson, Carson & Fisher, 2015; Roost et al., 2020). During the last decade, authors have gained interest on how the saddle configuration affects the rider’s performance. Keener et al. (2020) investigated how three different stirrup lengths affected peak acceleration on the rider’s back, concluding that shorter stirrups had the potential to reduce the impacts received by the rider.

Bike fitting (*i.e.*, adjusting bike shape and size to fit its rider’s ergonomic needs) has long been considered a very important tool for optimizing performance and reducing the risk of injury of those who practice in cycling disciplines (Fonda, Sarabon & Li, 2014; Ayachi, Dorey & Guastavino, 2015; Priego Quesada et al., 2017; Priego Quesada et al., 2019), because such sports require a particular posture of the cyclist to be sustained over long periods of time (Silberman, 2013). It would be reasonable to think that, in equestrian sports, adjusting saddle tilt (either by changing saddle dimensions and geometry, or by adding corrective saddle padding) and stirrups length to the rider’s anthropometrical and ergonomic needs could lead to similar benefits as bike fitting, since the points of contact of a cyclist with a bike are fundamentally the same as a rider with a horse (*i.e.*, the ischial tuberosities, the hands and the feet).

Therefore, we believe there is a gap in the literature that needs to be addressed: the saddle interface adjustments need to be understood as a link between both parts of the show jumping horse-rider dyad and not only for the animal as an independent being. Since the horse’s welfare during riding is affected by horse-rider harmony (Peham et al., 2001), and saddle design has a significant impact on the harmonious relationship of the show jumping dyad (White & Birkbeck, 2013) it can be assumed that optimizing saddle fit for the rider will ultimately have a positive impact on the horse’s welfare. The two main saddle set-up manipulations while tacking up a horse are stirrup length and saddle tilt. While there seems to be an agreement on seeking a saddle tilt of 0° (Dyson, Carson & Fisher, 2015; Roost et al., 2020), there is no clear evidence on how this affects the rider performance biomechanically. On the other hand, stirrup length varies depending on personal preference, skill and discipline: more experienced riders have been reported to select longer stirrup lengths when compared to novice riders during flatwork (Andrews-Rudd et al., 2018), which has been linked to a more developed independence of the seat (*i.e.*, better balance) of advanced riders. Generally, dressage riders will feel more comfortable on lower stirrups as they are required to perform sitting trot and specific exercises that will require them to sit deeper into the saddle, with close contact of their legs to the body of the horse (Farmer-Day et al., 2018). On the other hand, eventers, jockeys and showjumpers need to reduce the weight on the horse’s back and tend to choose higher stirrup lengths in order to achieve the so-called light-seat (Dumbell et al., 2016).

Literature objectively analysing issues related to a saddle not fitting the rider has previously been reported to be inexistent (Greve & Dyson, 2013), and to our knowledge there is still no scientific analysis of the interaction between saddle tilt and rider kinematics. However, directions to fit a saddle with a 0° angle seem of common agreement by the horse riding community (Bondi et al., 2020). Ideally, saddles should be manufactured to fit the horse and the rider, although horses change in shape and conformation over time (Harman, 1999; Greve & Dyson, 2015), and show jumping riders tend to use one saddle for more than a single horse (Greve & Dyson, 2015). Therefore, saddle pads and numnahs are a common solution that riders use, for their saddle to better fit the horse (Kotschwar, Baltacis & Peham, 2010). Several brands have developed correction pads which, by means of interchangeable shims of diverse materials, often foam, can alter the tilt of the saddle as opposed to regular pads which do not have the ability to be modified. While regular saddle pads and their impact on force distribution on the horse’s back has been already studied (Kotschwar, Baltacis & Peham, 2010), we are not aware of any scientific analysis of corrective pads.

Adjusting a saddle is dependent on a significant variety of factors, such as the singularities of each discipline, extrinsic factors like the subjective beauty of a stretched leg, coaches’ preconception of what a “good posture” should be, or the high level of inter-subject variability observed in the previously cited studies. As it occurs with bike fitting, there will always be controversy on how to adjust a saddle to fit a rider. Ultimately, there is not only one solution to this puzzle, because finding a solution requires individualization as no two riders are alike.

This study aims to describe how small adjustments on stirrup length and saddle tilt can alter attenuation of the shock waves that travel through the rider’s back and the tension force output on the stirrups and reins, both at posting trot and working canter. Our hypothesis is that every rider will respond differently to such adjustments, yet a trend will emerge, showing that shorter stirrups attenuate shockwaves better than longer stirrups, while tilting the saddle forward and backward will perturbate the ability of the rider to attenuate shockwaves. We also hypothesize that altering the shock attenuating ability will affect rider’s balance and ultimately the forces applied at the mouth of the horse (mouth piece) and stirrups. Consequently, we have added four tension loadcells (two stirrups and two sides of the mouth piece) that will monitor such mechanical consequences.

## Methods

### Participants

Eleven recreational show jumping riders (26 ± 8 SD years, training 2-4 h/week, 12 ± 5 SD years of experience) volunteered to participate in the study. Participants were asked to sign an informed consent after being informed of the study’s procedure. The National Medical Ethics Committee (Ministry of Health, Ljubljana) gave full approval to the project according to the declaration of Helsinki (approval number: 0120-99/2018/5).

One Koninklijk Warmbloed Paard Nederland (K.W.P.N.) school-type horse (9 years), jumping up to 1.00-m shows was used for the study. The height of the horse was 162 cm (from the ground to the withers) and the weight was 550 kg. It was sound and supple, and showed self-carrying capacities. A veterinarian checked the animal and declared it was able to withstand the demands of the study design.

### Experimental protocol

All measures were taken at posting trot and working, 3-point position canter, which are gaits that showjumpers are familiar with.

**Figure 1:**
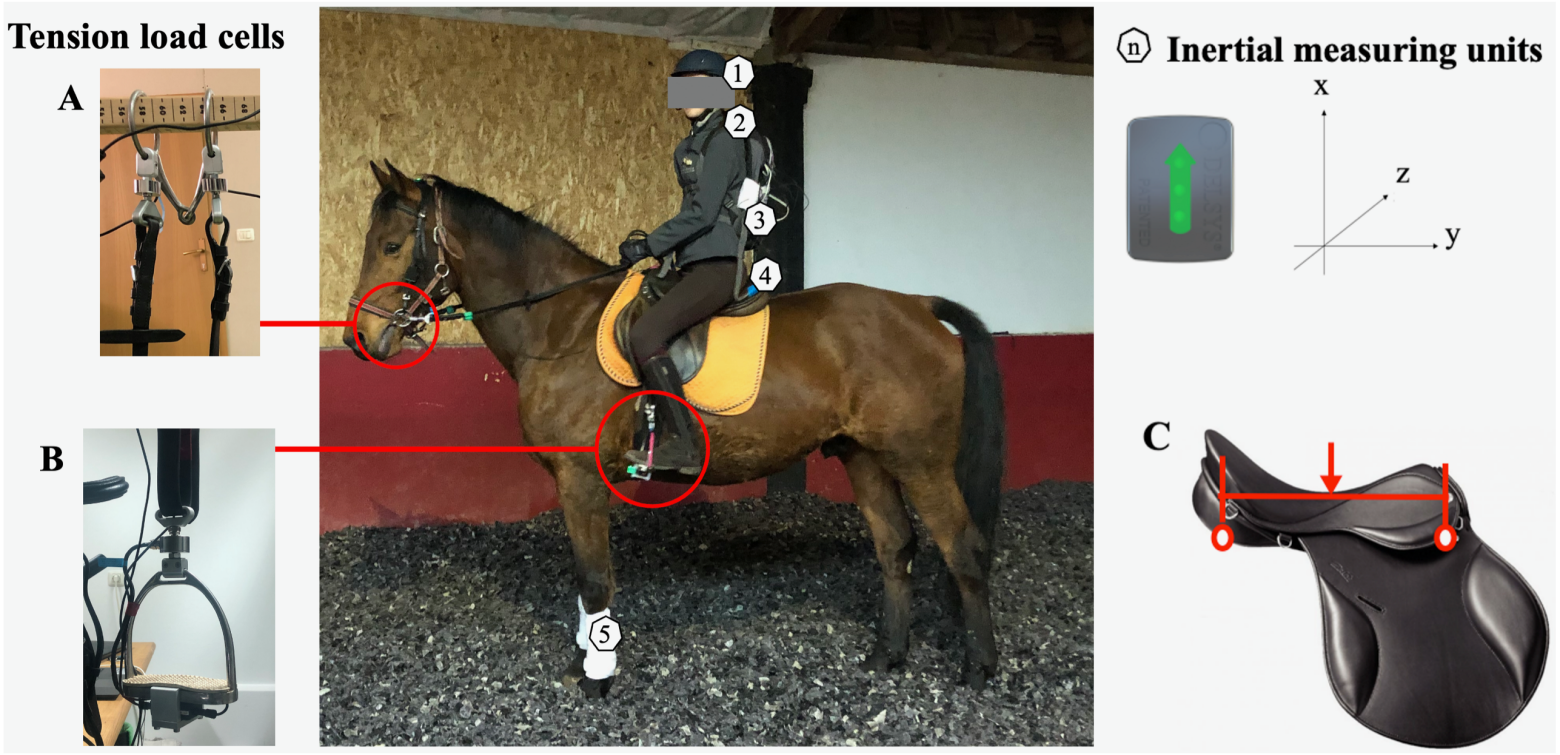
Placement and orientation of the tension loadcells of the bridles (A) and stirrups (B) and the inertial measuring units (IMUs). (1) Helmet, (2) 7th cervical vertebrae, (3) anterior sacral promontory, (4) cantle of the saddle, (5) forelimb of the horse. (C) Close-up picture of the saddle at a neutral position. Two circles indicate the most distal ends of the saddle in contact with the horse’s back. Arrow indicates the lowest part of the seat, which was aimed to be at the center between the two most distal ends, with the horse at halt and squared.

A repeated measures, single visit study design was implemented. All measures lasted <2 h and took part on a 20 × 40 m geotextile footed indoor arena. After obtaining informed consent of the participants, thirteen inertial measuring units (IMUs) (Delsys Trigno, Natick, MA, USA) were placed on the rider, saddle, bridles and horse (Fig. 1). Five trials were performed: (1) one standardization trial, (2) one trial with short stirrups, (3) one trial with long stirrups, (4) one trial saddle. Trials 2–5 were randomized. On the standardization trial (refer to ‘Horse set-up’ for details of the saddle setup), 120 strides on the right hand and 120 strides on the left hand were recorded at trot and canter while the horse was being ridden around the arena. On the remaining trials, 60 strides on the right hand and 60 strides on the left hand at trot and canter were recorded (refer to ‘Interventions design’ for details of the saddle setup). Participants were encouraged to keep the horse balanced and straight at all times.

The horse was cooled down at walk on loose reins upon completion of all five trials. The horse had a >24-h rest between trials.

### Horse set-up

A level II national accredited coach (Real Federación Hípica Española) set up the horse and performed all the interventions. An all-purpose, 18″saddle (Zaldi Royal, Zaldi, Salamanca, Spain) was used. The saddle was initially fitted with a 0°angle. On a flat, rigid surface, and with the horse at halt and squared, the deepest part of the seat was positioned in the middle between the most distal points of contact between the saddle and the horse’s back, in the antero-posterior plane (Fig. 1), following the standardized agreements proposed elsewhere (Roost et al., 2020). Stirrup leather length was established by asking the riders to sit comfortably and relaxed, with regular riding posture and then the length of the stirrup leathers was adjusted to achieve a knee angle as close as possible to 90° when stationary. Knee angle was measured using a virtual photometric goniometer (Milanese et al., 2014). The bridle was assembled with a simple snaffle bit. Noseband pressure was checked with a commercially available noseband pressure gauge (International Society for Equitation Science, Australia).

Both forelimbs of the horse were covered with a schooling bandage, with one embedded IMU in each limb. The stirrups and the rings of the bit were equipped with a tension load cell.

### Interventions design

From the neutral position, stirrups were either lowered or lifted by 60 mm, which corresponded to 3 holes and is considered to elicit a noticeable change of feel for the rider (Hyun & Ryew, 2015). For the saddle tilting trials, a commercially available corrector saddle pad (PE correction saddle pad, Premier Equine International Ltd., United Kingdom) was used to tilt the saddle forward or backward 4° without the rider, which is the maximum correction that the saddle pad allows.

### Data logging

#### Force measures

Tension forces at the end of the reins and stirrup leathers were registered. The mouth bit was equipped with a uniaxial tension load cell (FL25-5 kg Forsentek Co., Limited, Shenzhen, China) on each ring, aligned with the direction of the reins. Stirrups were equipped with a uniaxial tension load cell (FL35-200 kg Forsentek Co., Limited, Shenzhen, China) on the top groove, aligned with the stirrup leather (Fig. 1). Sensors were cabled to a single-channel amplifier (INSAmp, ISOTEL, Logatec, Slovenia) and routed to a data logger through a multichannel acquisition card (NI USB-6218, National Instruments, Austin, TX, USA). The data logger and acquisition card were embedded on a lightweight, tight immobile backpack, mounted on the rider. Force output was recorded at a sampling rate of 1,000 Hz, through a custom-made software (S2P d.o.o., Slovenia) developed with LabView 2015 (National Instruments, Austin, TX, USA). A 3-point calibration was used for the sensors, with known weights of 10 kg, 15 kg and 20 kg for the snaffle sensors and 40 kg, 100 kg and 150 kg for the stirrup sensors.

#### Inertial measures

Thirteen wireless IMUs (Delsys Trigno; Delsys, Monmouth Junction, NJ, USA) were placed on the horse-rider dyad (Fig. 1). Specifically, three IMUs were mounted on the rider, one at each of the following locations: helmet, spinous process of the 7th cervical vertebrae (C7) and anterior sacral promontory (S1). Three additional IMUs were placed on the cantle of the saddle and the horse’s forelimbs. Raw signal was amplified (gain: 2000) and recorded at a sampling rate of 150 Hz with the built-in amplifier. Acceleration from the IMUs was used for the calculation of shock attenuation, with an accelerometer output of a range of ±16 g (Delsys Trigno; Delsys, Monmouth Junction, NJ, USA).

#### Synchronization of signals

A trigger module was configured such that the Delsys Trigno IMU system worked as the master that enslaved the custom-made software that registered the force output. At initiation of each trial, a trigger impulse was sent by the Trigno base, which started all recordings. During the experimental protocol, the trigger was used to delimitate initiation and finalisation of condition bouts, which did not affect synchronization.

### Data analysis

All signals were processed ex situ using MATLAB R2020b (Natick, MA, USA). Each condition bout was identified with the signals of the trigger module, and acceleration and force values were averaged across each condition.

**Shock attenuation (SA)** was measured between the resultant accelerations of the helmet and saddle (helmet:saddle) and the 7th cervical vertebrae and anterior sacral promontory (C7:S1). SAs were calculated using a transfer function (Castillo & Lieberman, 2018) given in decibels (dB): *SA = 10Log10(ACChigh/ACClow)*

ACChigh and ACClow represent, respectively, the power spectral densities of the accelerations recorded by the highest and lowest positioned accelerometers with regards to the vertical plane. The lowest accelerometer’s data are placed at the denominator so that negative values represent attenuation, whereas positive values indicate an increase in signal strength.

**Force output** was registered from the rein and stirrup tension loadcells. Forces below the 5th percentile and above the 95th percentile were not included in the analysis as they can be assumed to be generated by factors not directly related to the riding technique such as the horse stumbling, pulling on the reins or even coughing (Eisersiö et al., 2015) that could lead to either an abnormally high force output or a loss of contact with the sensors. Following the elimination of extraneous events, each condition bout was averaged including right and left forces. A symmetry index (SI) between right and left rein sensors and stirrup sensors was calculated as a percentage by dividing the subtraction of mean force of the right - left sensors by the highest mean force between right and left sensors as follows:

\begin{eqnarray*}\text{Symmetry index}= \frac{\text{Right sensor mean force}-\text{Left sensor mean force}}{\text{Maximum of left and right mean force}} . \end{eqnarray*}

Dominance of the right/left limbs was assumed to be as follows: left dominant SI ≤ −0.05; right dominant SI ≥ −0.05, neutral or no dominance −0.05 <SI >0.05.

### Statistical analyses

Statistical analyses were performed using SPSS (IBM SPSS Statistics for Windows, Version 27.0. Armonk, NY: IBM Corp). Both types of interventions (saddle tilt and stirrup length adjustments) were analysed with repeated measures ANOVA which were conducted on shock attenuation, absolute force output and symmetry index of the stirrups and reins. Each condition (neutral/long/short stirrups and neutral/forward/backward saddle tilt) were treated as repeated measures. *Post-hoc* pairwise comparisons within repeated measures were performed at an alpha level of 0.05.

Normality was checked through visual inspection of the residuals plots as suggested elsewhere (Kozak & Piepho, 2018), and the normality criteria were met. Effect size was calculated as *η*^2^ and interpreted as large (.14), medium (.06), and small (.01) effects (Fritz, Morris & Richler, 2012).

## Results

### Shock attenuation

**Figure 2:**
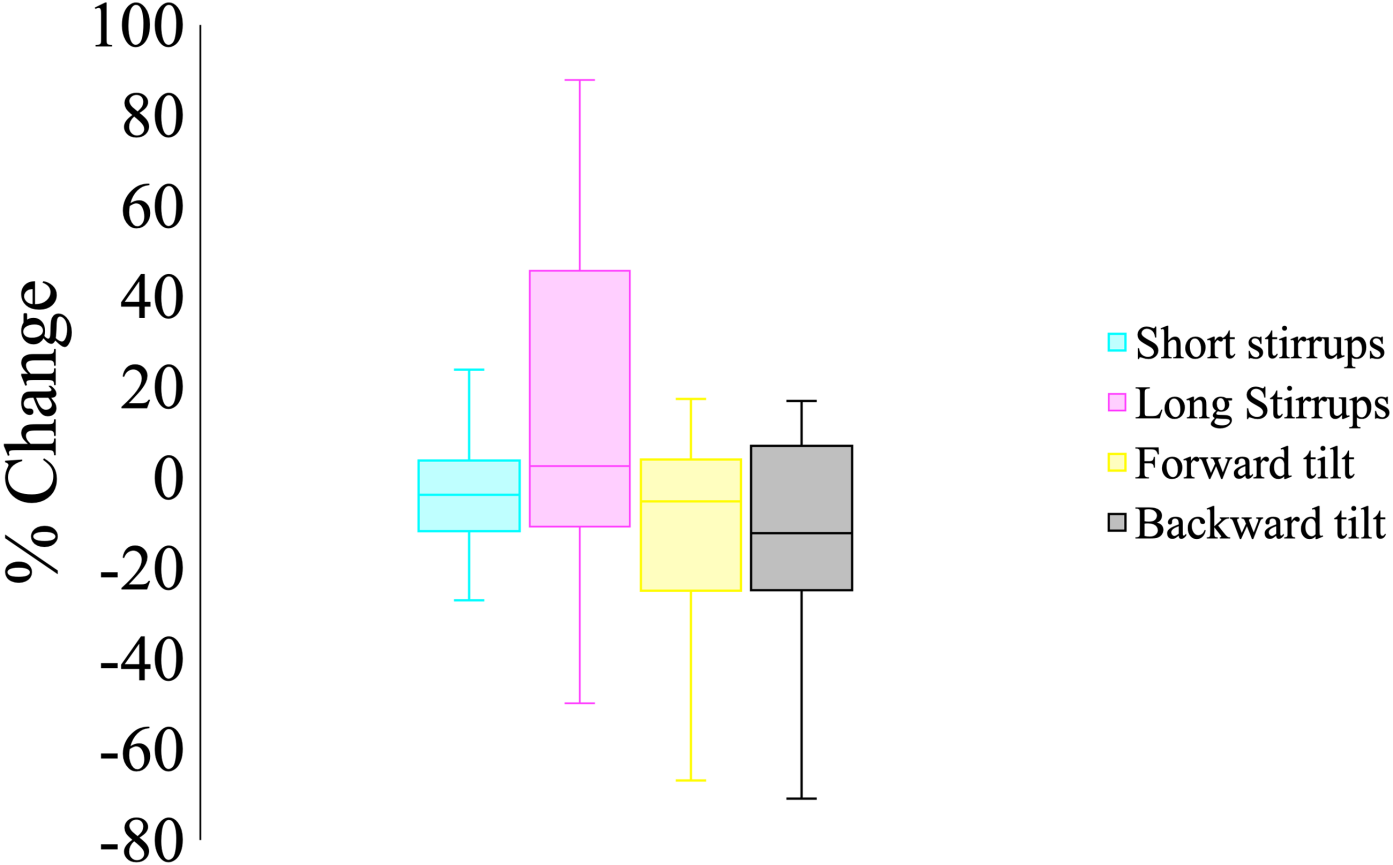
Boxplot of the percent change of helmet:saddle shock attenuation waves in each condition compared to the standardization trial in canter. Shortened stirrups had a greater contribution to attenuating shockwaves compared to longer stirrups (*p* < 0.05) and forward and backward tilting the saddle also enhanced shock attenuation (*p* < 0.05), although no difference was observed between the two saddle setups (*p* = 0.19).

A repeated measures ANOVA revealed a statistically significant interaction across conditions on the helmet:saddle SA waves in canter (F(4,48) = 4.852, *p* = 0.02, *η*^2^ = 0.3). Figure 2 summarizes the percent change difference between the standardization trial and each intervention at canter on the helmet:saddle SA waves. *Post-hoc* analyses showed that shortened stirrups had a greater contribution to attenuating shockwaves compared to longer stirrups (*p* < 0.05) and forward and backward tilting the saddle also enhanced shock attenuation compared to neutral tilt (*p* < 0.05), although no difference was observed between the two saddle setups (*p* = 0.19). No significant main effects were observed across conditions on the helmet:saddle waves in trot nor the C7:sacrum waves in trot and canter.

**Figure 3:**
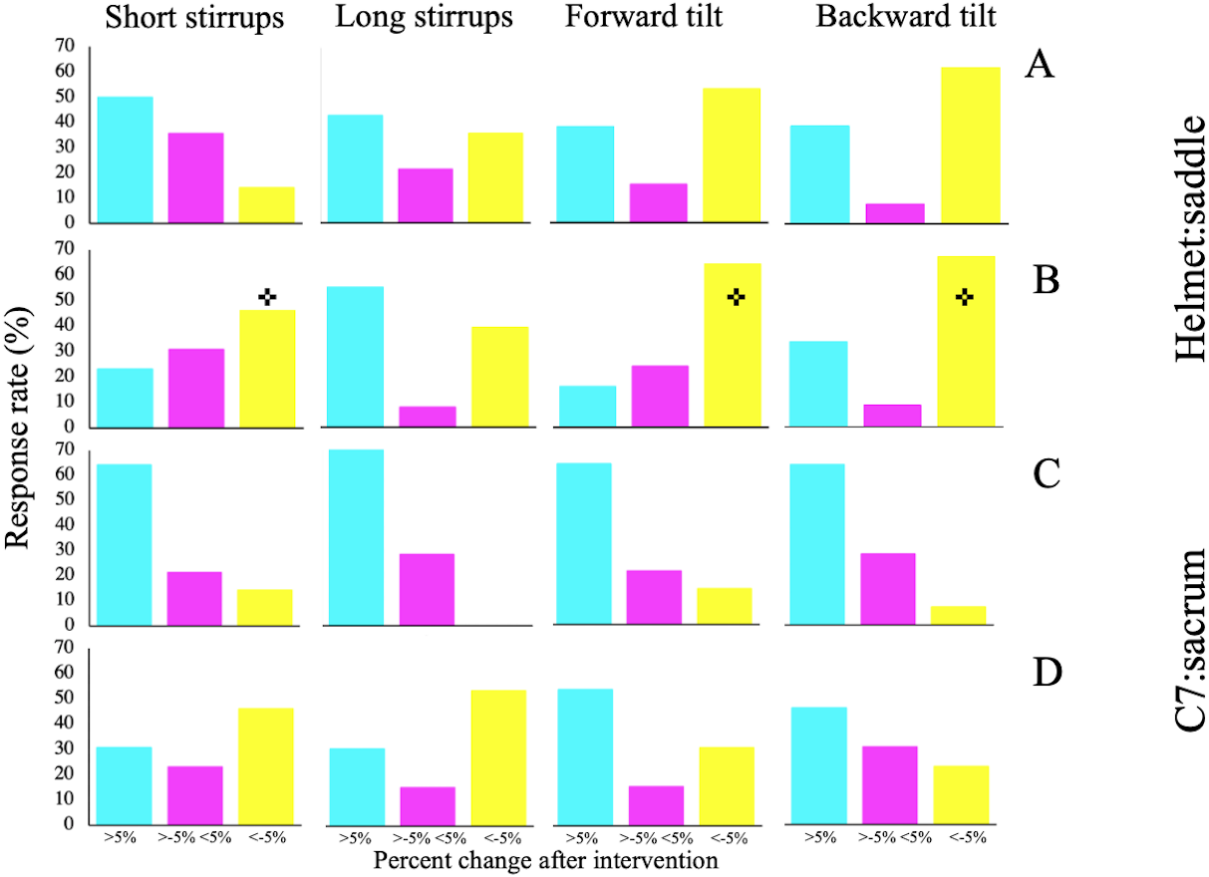
Percentage of subjects that responded negatively (>5%), did not respond (≥5% <5%) or responded positively (≤5%) to each intervention on (A) helmet:saddle at trot, (B) helmet:saddle at canter, (C) C7:sacrum at trot and (D) C7:sacrum at canter. Please note that a positive response leads to a negative change. Refer to Castillo & Lieberman (2018) for more information on the interpretation of shock attenuation waves. +Significantly optimized shock attenuation waves.

A very high inter-subject variability on the response to the saddle set-up modifications was observed on all indices of shock attenuation. Figure 3 depicts the amount of respondent and non-respondent subjects, and the direction of response based on individual percent change from the standardization trial to each saddle modification.

#### Absolute force output

**Figure 4:**
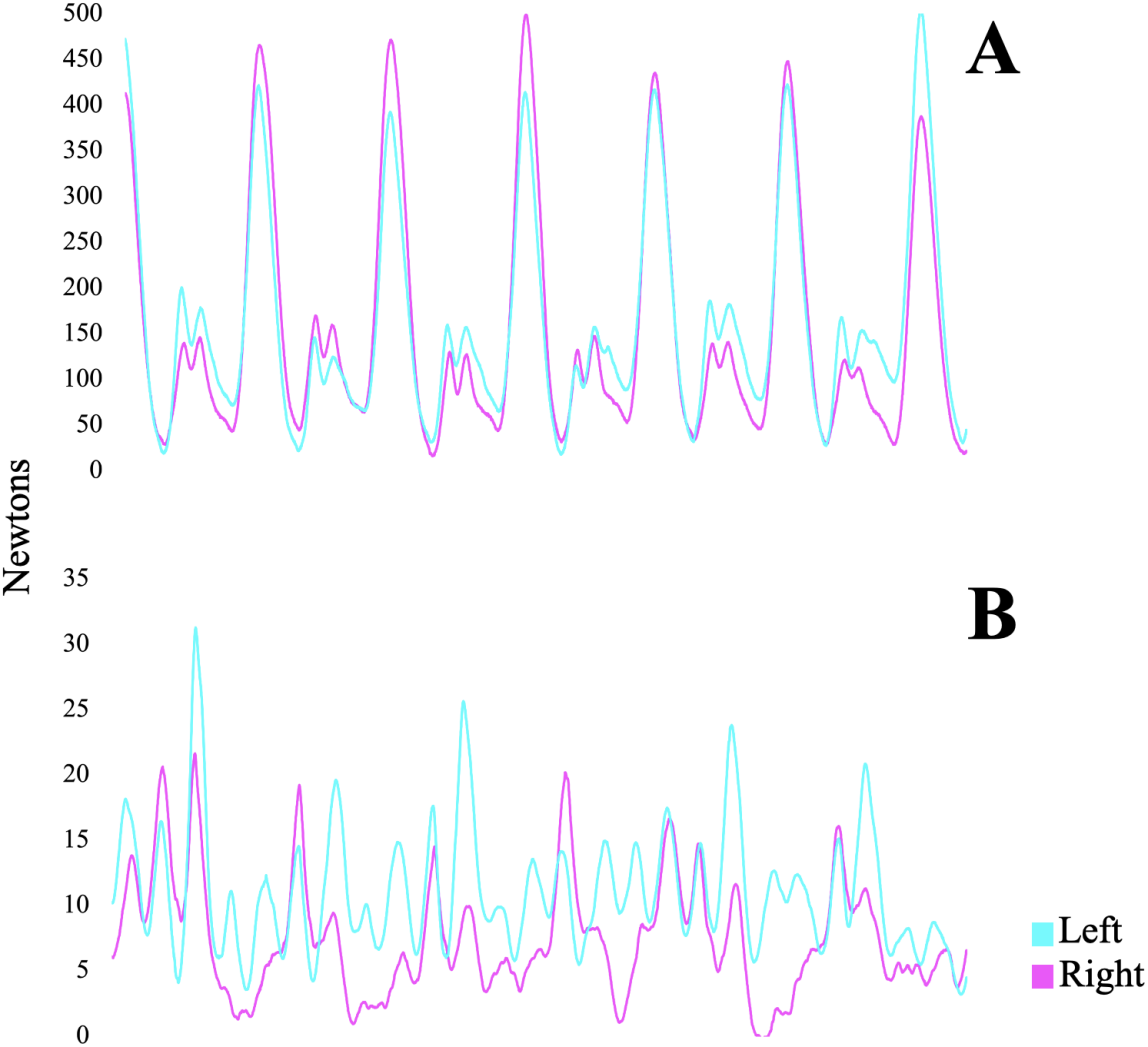
Raw signals from a representative subject of (A) stirrup forces and (B) rein forces at trot over seven strides.

**Figure 5:**
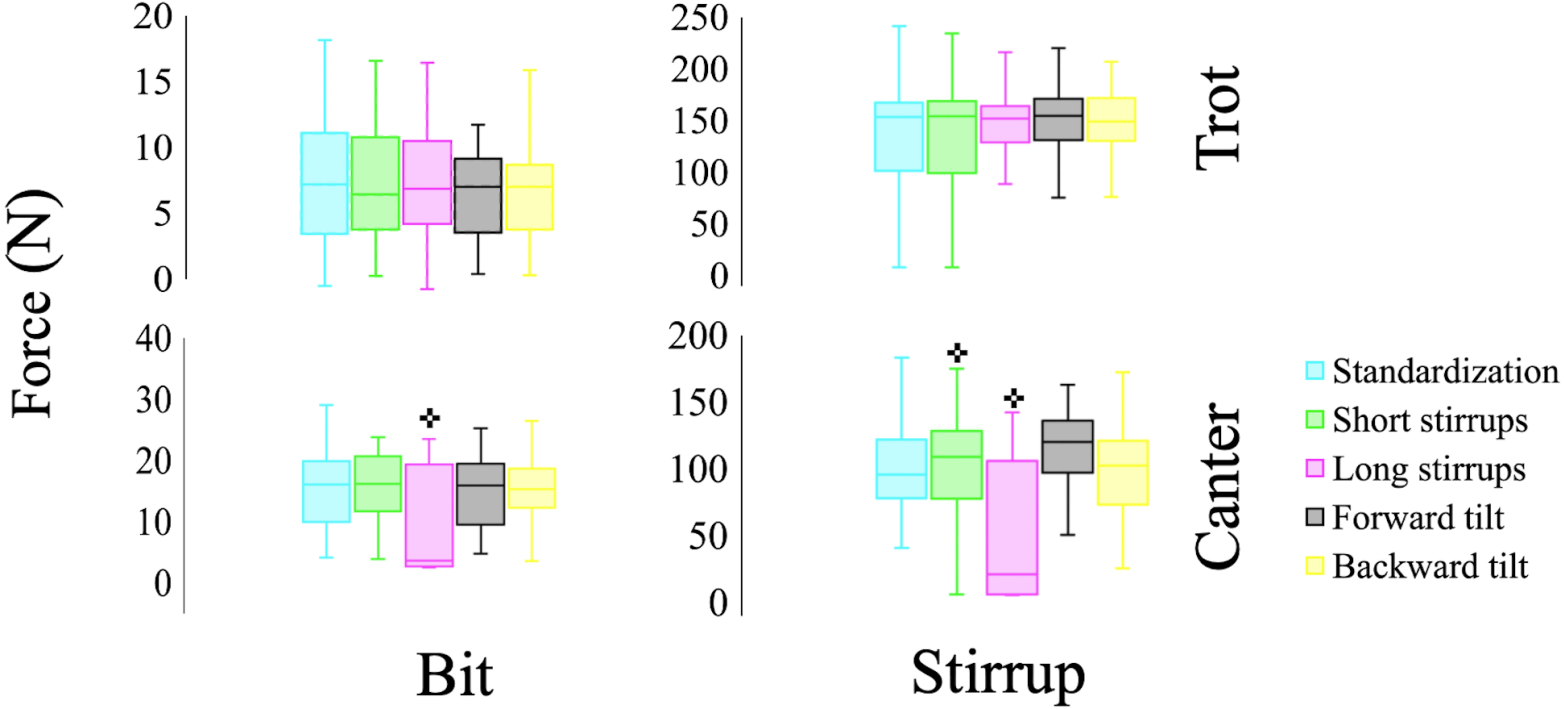
Summaries of absolute force outputs at the stirrups and bit during trot and canter in each condition. +Significantly different from standardization trial.

Figure 4 shows a representative sample of raw data from the stirrups and rein tension loadcells at trot over a period of seven horse strides. A repeated measures ANOVA revealed a significant interaction in absolute force output at the bridle sensors in canter (F(4,156) = 11.662, *p* = 0.001, *η*^2^ = 0.2) and at the stirrup sensors in canter (F(4,156) = 2.852, *p* = 0.02, *η*^2^ = 0.02) (Fig. 5). *Post-hoc* analysis showed lower rein tension forces when stirrups were lowered at canter (*p* < 0.01), as well as an increase in stirrup forces with shorter stirrups (*p* < 0.05 and a decrease in stirrup forces with longer stirrups (*p* = 0.01). Table 1 shows the average force output registered at the bit and stirrups.

### Symmetry index

**Figure 6:**
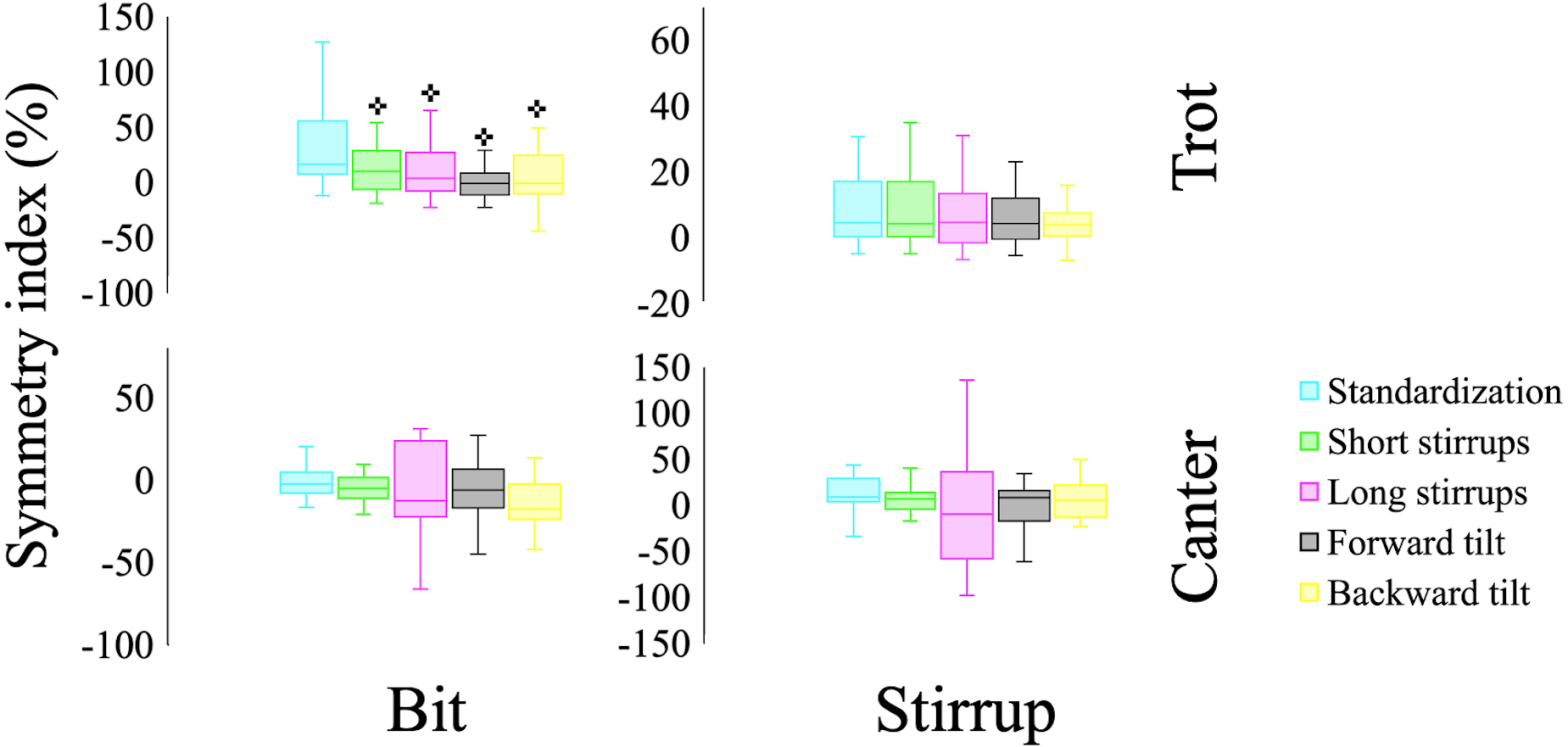
Summaries of force symmetry index at the stirrups and bit during trot and canter in each condition. +Significantly different from standardization trial.

A repeated measures ANOVA revealed a significant interaction in force symmetry index at the bridle tension load meters in trot (F(4,76) = 4.748, *p* = 0.02, *η*^2^ = 0.2). Leg symmetry index had no significant changes in either condition. A *post-hoc* analysis revealed that all conditions significantly decreased symmetry index at the bridle sensors in trot (*p* < 0.05). Figure 6 summarizes the symmetry index outcomes. In trot, the bridle tension load meters showed that 90% of the subjects were right dominant and 10% were neutral. However, dominance suffered drifts throughout the interventions (Table 2).

**Table 1:** Average force output of the stirrups and bit.

		**FORCE (N)**																
		Standardization			Short stirrups				Long stirrups				Forward tilt			Backward tilt		
		Mean		(SD)	Mean		(SD)		Mean		(SD)		Mean		(SD)	Mean		(SD)
Trot	Bit	8.06	±	4.36	7.91	±	4.15		7.93	±	3.84		7.68	±	3.89	7.49	±	3.64
	Stirrup	132.51	±	54.79	133.93	±	51.16		182.87	±	213.28		188.39	±	212.14	187.65	±	211.90
	Canter	Bit	14.39	±	7.59	14.63	±	6.56		8.31	±	9.46	^*^	14.11	±	6.32	14.20	±	7.29
Stirrup		92.94	±	38.10	100.99	±	39.59	^*^	74.18	±	172.14	^*^	157.90	±	217.71	144.36	±	221.52

**Notes.**

SD, standard deviation.

* Significantly different from standardization trial.

**Table 2:** Lateral dominance of the sample group. Percentages have been calculated based on the symmetry index on the following basis: left dominant SI ≤ −0.05; right dominant SI ≥−0.05, neutral or no dominance −0.05 < SI > 0.05.

	Trial				
Lateral dominance	**Standardization**	**Short stirrups**	**Long stirrups**	**Forward tilt**	**Backward tilt**
Right	80%	50%	50%	20%	50%
Left	0%	30%	20%	40%	50%
Neutral	20%	20%	30%	40%	0%

## Discussion and Implications

This study has investigated the effects of three different saddle tilts and stirrup lengths on the shock attenuation of show-jumping riders’ spine, the forces at the bit and the forces at the stirrups. We expected singular and distinct responses from each rider, while depicting common trends. Briefly, we hypothesised that better attenuation would be achieved with shorter stirrups and a neutral saddle tilt. However, our hypothesis was not always met.

We observed that shortening the stirrups enhanced shock attenuation while riding at canter and increased the force applied at the stirrups both at trot and canter. Longer stirrups lead to lighter stirrup forces. Interestingly, the overall force applied to the stirrups has a direct transfer to a similar force underneath the saddle (van Beek et al., 2012). However, these results agree with previous findings and hypotheses which suggest that peak forces underneath the saddle decrease with higher stirrup forces (de Cocq et al., 2010; Peham et al., 2010; van Beek et al., 2012).

It has previously been suggested that short stirrup lengths are associated with higher incidence of low back pain, although results have been inconclusive (Quinn & Bird, 1996). More recent studies have demonstrated that the key to reducing back pain is achieving an adequate attenuation of the shockwaves transmitted from the horse to the lumbopelvic hip complex through the saddle (Cejudo, Ginés-Díaz & Sainz de Baranda, 2020). It has been observed that showjumpers tend to select shorter stirrup lengths in order to better absorb the shocks generated when landing jumps (Pugh & Bolin, 2004), and it has been suggested that riders with back pain can counteract it with lower stirrup lengths (Keener et al., 2020), but no evidence has yet been provided to clearly describe how longer or shorter stirrups influence shock attenuation. Our data support such observations since it shows an optimised shock attenuation and an increase on stirrup forces with shorter stirrups. These changes mean that there is a drift of impact origin from the saddle to the stirrups, and such a drift allows more of the rider’s joints to act during shock absorption and body balance mechanisms. On the other hand, it is important to bear in mind that riding with short stirrup lengths may cause a pelvic retroversion due to a more closed hip angle, which can reduce lumbar lordosis (Cejudo, Ginés-Díaz & Sainz de Baranda, 2020), increase tension on the posterior ligaments of the lumbar spine (Pizones & García-Rey, 2020), and potentially lead to injuries, particularly in riders with previous pathologies.

Adjusting saddle tilt either posteriorly or anteriorly resulted in an improved shock attenuation at canter, suggesting that small (<4°) antero-posterior tilts can significantly reduce impact on the rider’s back, with 65% of the subjects responding positively to such saddle modifications. Nicol et al. (2014) proposed that enhancing the saddle’s panels shock attenuating characteristics had a negative impact for the rider, arguing that riders had a “diminished feel of the horse below them”. This diminished feel led them to take pressure off the stirrups and bear their weight on the saddle. It could be argued that the riders in our study showed altered shock attenuating levels due to changes in the awareness of the horse after manipulating saddle tilt. However, contrary to the proposed hypothesis, our results resulted in decreased shock transmission and no changes in stirrup absolute forces after saddle tilt adjustments.

At trot, adjusting saddle tilt and stirrup length surprisingly decreased symmetry index of forces applied to the bit, likely because riders were more focused on their body weight adapting to their newly adopted posture. Table 2 shows how most riders were initially either neutral or right dominant, and suffered a drift throughout the trials. It is worth noting the high level of inter-subject variability, which highlights the importance of individualization.

A neutral saddle tilt of 0° has been recommended to avoid saddle slip and rider unbalance (Bondi et al., 2020), although specific tilt angle ranges that can generate such undesirable outcomes have not been clearly specified, making it difficult to contrast the existing data with our results. From the figures of the published research, one can presume that such angles are >8°, which are double the magnitude of the angles used in the present study’s interventions.

## Conclusion and Practical Implications

In any athletic discipline it is hard to postulate absolutes in terms of correct techniques, equipment setups, *etc*. In equestrian sports it is even harder, as a living animal that we barely understand comes into the game as the main character. The findings that have been presented in this manuscript can serve as a base to modifying specific parameters of the saddle setup according to the needs of each rider. In the present study, two saddle set-up modifications that horse riders can perform have been analysed and discussed, and while some conclusions can be drawn, at this stage it would be premature to claim a correct way of adjusting saddle parameters to fit a rider. It is undeniable however, that the importance of individualization is paramount, and that adjusting a saddle to fit a rider has a potential impact on the rider’s back health and should not be neglected. Moreover, our data suggests that elongating stirrups shifts the weight from stirrup to saddle and shortening does the opposite action. While no length is better than another, it is useful to know the direction of change to properly apply it during training and competition in a show-jumping setting.

## Limitations

A potential concerning limitation is the use of a single horse. However, we selected such a design to reduce any variability that could be introduced by using several horses. Care should be taken when interpreting these results, as a substantial part of the group responded either positively or negatively to the interventions. Please note that a positive response leads to a negative change. Refer to Castillo & Lieberman (2018) for more information on the interpretation of shock attenuation waves. The horse feeling a different motion on the different experimental set-ups can also play a role in variability, since it might be displaying a different back motion in each condition. The use of commercially available equipment has also limited the refinement of the results. For instance, achieving saddle tilt with correction saddle pads will not lead to the same results as modifying the saddle design so that the panels remain aligned while only the seat tilts for the rider. This is why individualization of saddle setup must be performed with care and by using the general agreements on saddle tilt and stirrup lengths proposed elsewhere (Kotschwar, Baltacis & Peham, 2010; Andrews-Rudd et al., 2018; Farmer-Day et al., 2018; Keener et al., 2020; Roost et al., 2020) only as a starting point. From that starting point, one must reach the optimal setup through trial and error.

## Additional Information and Declarations

### Data Availability

The following information was supplied regarding data availability: The raw data is available at Zenodo: Elmeua, Marc, & Sarabon, Nejc. (2022). Dataset: Effects of saddle tilt and stirrup length on horseback rider’s performance [Data set]. Zenodo. https://doi.org/10.5281/zenodo.6417358.
